# Generation of cell-type-specific gene mutations by expressing the sgRNA of the CRISPR system from the RNA polymerase II promoters

**DOI:** 10.1007/s13238-015-0169-x

**Published:** 2015-06-07

**Authors:** Jiaqiang Wang, Xin Li, Yanhua Zhao, Jingyu Li, Qi Zhou, Zhonghua Liu

**Affiliations:** College of Life Sciences, Northeast Agricultural University, Harbin, 150030 China; State Key Laboratory of Reproductive Biology, Institute of Zoology, Chinese Academy of Sciences, Beijing, 100101 China

**Dear Editor,**

Recently, the CRISPR/Cas9 system is emerging as a powerful tool for genome editing (Chang et al., 2013; Li et al., 2013; Niu et al., 2014; Shen et al., 2013; Wan et al., 2015; Wang et al., 2013) and genetic screening (Konermann et al., 2015), and holds great promise for biomedical applications in disease modeling and gene therapy by *in vivo* genome editing (Maddalo et al., 2014; Xue et al., 2014). However, the applications of CRISPR/Cas9 system still face some technical hurdles, one of which is to harness the gene editing in a precisely controlled manner. Of the two-component CRISPR/Cas9 system for genome editing, the Cas9 is a fixed genome-cutting component expressed from the RNA polymerase II (pol II) promoter that can drive tissue-specific gene expression; while the single-guide RNA (sgRNA) is a changeable genome-guiding component expressed from RNA polymerase III (pol III) promoter that usually drives the ubiquitous expression of “housekeeping” genes in all tissues. Therefore, to express the sgRNA in a tissue-specific manner can provide a convenient approach to tissue-specific gene mutations. Here, we reconstructed the sgRNA to enable its expression from the pol II promoters, and further achieved cell-type specific gene mutations via the modified CRISPR/Cas9 system by using cell-type specific pol II promoters-driving sgRNA.

To generate pol II promoter-driving sgRNAs, we constructed a microRNA-shRNA-embedded sgRNA (miRsh-sgRNA) cassette that could express the small RNA from pol II promoter (Wang et al., 2007) into the 3′-untranslational region (UTR) of the DsRed reporter gene (Figs. 1A and S1, and Supplementary Materials), as methods by cis-acting ribozymes (Gao & Zhao, 2014; Nissim et al., 2014) and Cas6/Csy4-based RNA processing (Nissim et al., 2014) have been reported. Notably, the nonsense shRNAs and a reported efficient sgRNA targeting the mouse *p53* gene (sgp53) (Xue et al., 2014) was adopted in the construct for a proof-of-concept experiment. The mature sgRNA derived from the miRsh-sgRNA cassette will have additive 7 nucleotides in the 5′-end (Fig. 1A), so we chose a reported optimized sgRNA backbone, the sgRNA^(F+E)^, to improve the mutagenesis efficiency (Chen et al., 2013), given that the sgRNAs with mispairing and addition in the 5′-end are still functional (Cong et al., 2013).

**Figure 1:**
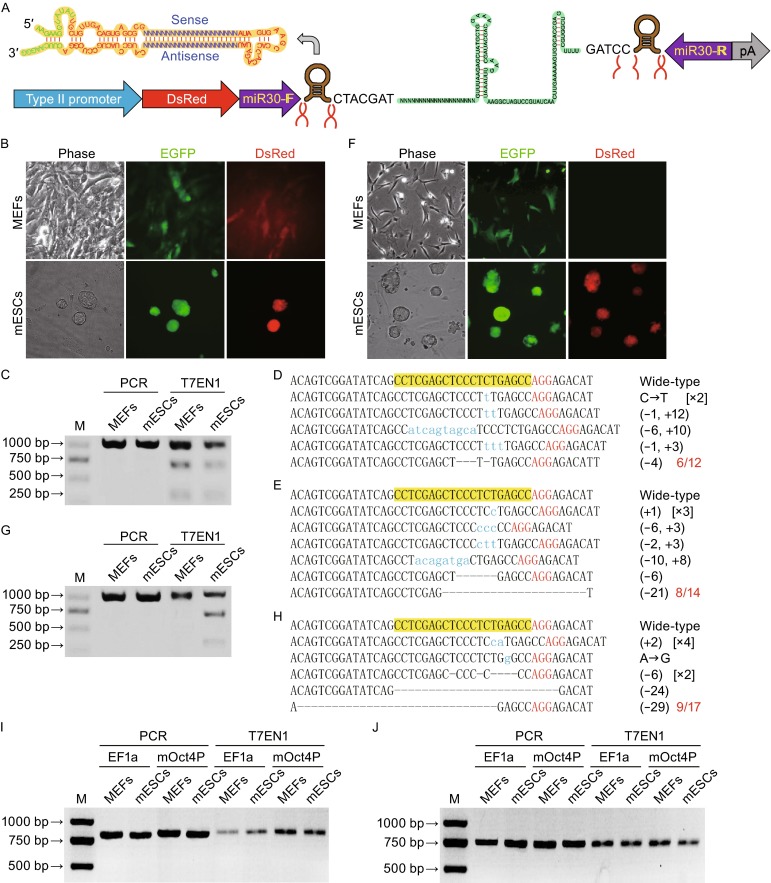
**Pol II promoter-driving miRsh-sgp53 functions in MEFs and mESCs**. (A) Structure of pol II promoter-driving miRsh-sgRNA cassette. The cut sites of Drasha in this primary-microRNA are marked, and the resulted sgRNA would have 7 additive nucleotides (CTACGAT) at the 5′-end. (B) MEFs and mESCs transfected with Cas9-EGFP and constitutive EF1a promoter-driving DsRed-miRsh-sgp53 vectors are both GFP and DsRed positive at 48 h after transfection. (C) T7EN1 cleavage assay showed that constitutive EF1a promoter-driving miRsh-sgp53 can guide Cas9 for producing DSBs in both MEFs and mESCs. The PCR band is 972 bp and the theoretical cut bands are 286 bp and 686 bp. (D and E) Representative Sanger sequencing results of the PCR amplicons from MEFs (D) or mESCs (E) transfected with EF1a-driving miRsh-sgp53 cassette. AGG (red) is the protospacer-adjacent motif (PAM) sequence. Mutations were described in brackets. (F) MEFs transfected with Cas9-EGFP and stem cell-specific mOct4P promoter-driving DsRed-miRsh-sgp53 vectors are only GFP positive while mESCs are both GFP and DsRed positive at 48 h after transfection. (G) T7EN1 cleavage assay showed that stem-cell-specific mOct4P promoter-driving miRsh-sgp53 can guide Cas9 for producing DSBs only in mESCs but not in MEFs. The PCR band is 972 bp and the theoretical cut bands are 286 bp and 686 bp. (H) Representative Sanger sequencing results of the PCR amplicons from mESCs transfected with mOct4P-driving miRsh-sgp53 cassette. AGG (red) is the protospacer-adjacent motif (PAM) sequence. Mutations were described in brackets. (I) Off targeting analysis of p53-off-27 site in MEFs and mESCs transfected with EF1a-driving miRsh-sgp53 and Cas9 or mOct4P-driving miRsh-sgp53 and Cas9 by T7EN1 assay. The PCR band is 800 bp and the theoretical cut bands are 315 bp and 485 bp. (J) Off targeting analysis of p53-off-28 site. The PCR band is 753 bp and the theoretical cut bands are 281 bp and 472 bp

To test whether functional sgRNA can be efficiently derived from the pol II promoter-driving miRsh-sgRNA cassette, we transfected mouse embryonic fibroblasts (MEFs) and mouse embryonic stem cells (mESCs) with constitutive EF1a promoter-driving miRsh-sgp53 expression vector and Cas9-P2A-EGFP expression vector. GFP and DsRed double positive cells were sorted by fluorescence-activated cell sorting (FACS) two days after transfection for further analysis (Fig. 1B). The T7EN1 cleavage assay of these cells showed that constitutive EF1a promoter-driving miRsh-sgp53 can guide Cas9 for producing double strand breaks (DSBs) in *p53* gene in both MEFs and mESCs (Fig. 1C). To confirm the T7EN1 cleavage results, Sanger sequencing was performed and the result showed that the efficiency of miRsh-sgp53 in MEFs (Fig. 1D) and mESCs (Fig. 1E) was about 50% (6/12) and 57.1% (8/14). These results indicated that the functional sgRNA could be expressed from the pol II promoter-driving miRsh-sgRNA construct for successful gene mutations.

Further, to examine whether the miRsh-sgRNA cassette can produce gene mutation in a cell type-specific manner, we constructed an expression vector using the embryonic stem cell-specific mouse *Oct4* gene promoter (mOct4P) to express the miRsh-sgp53 cassette. Two days after transfection of MEFs and mESCs with this vector and the EF1a promoter-driving Cas9-P2A-EGFP vector, GFP and DsRed double-positive mESCs were observed, but only GFP positive MEFs were observed, indicating the cell-type-specific expression of the sgRNA (Fig. 1F). We sorted GFP and DsRed double positive mESCs and GFP positive MEFs by FACS for further analysis. T7EN1 cleavage assay and Sanger sequencing were performed, and *p53* gene mutation was only detected in the mESCs but not in the MEFs (Fig. 1G). The result of Sanger sequencing showed that the *p53* mutation efficiency in mESCs with ESC-specific mOct4P-driving miRsh-sgp53 (52.9%, 9/17) was similar to that with constitutive EF1a promoter-driving sgp53 (Fig. 1H). These results indicated that the cell type-specific gene editing can be achieved by cell type-specific promoter-driving expression of miRsh-sgRNA.

Previous studies suggested that CRISPR/Cas9 system would probably induce off-target mutations because the binding to genome could tolerate sequence mismatches distal from the PAM at the 5′ end of sgRNAs. It has been demonstrated in bacteria and cultured human cells that the DNA cleavage specificity of CRISPR/Cas9 system is determined by the PAM sequence NGG and the 8–12 base ‘‘seed sequence’’ at the 3′ end of the sgRNA (Cong et al., 2013; Jinek et al., 2013; Wang et al., 2013). We searched for potential off target sites based on this rule, and found 41 potential off targets of the sgp53 (named p53-off-1 to p53-off-41) existing in mouse genome (Table S2). The T7EN1 assay and Sanger sequencing revealed that no potential off target site we tested (Figs. 1I, 1J and S2) was mutated by miRsh-sgp53 in all the examined cells.

In conclusion, we designed a new construct for efficient and visible expression of sgRNAs from the pol II promoters, which therefore can produce cell-type specific mutations. This reconstructed pol II promoter-driving miRsh-sgRNA backbone will make the CRISPR/Cas9 system-mediated genome editing be more controllable and safer for future applications such as in *in vivo* gene therapy.

## Electronic supplementary material

Supplementary material 1 (PDF 958 kb)
