# Erratum to: Generation of cell-type-specific gene mutations by expressing the sgRNA of the CRISPR system from the RNA polymerase II promoters

**DOI:** 10.1007/s13238-015-0215-8

**Published:** 2015-09-28

**Authors:** Jiaqiang Wang, Xin Li, Yanhua Zhao, Jingyu Li, Qi Zhou, Zhonghua Liu

**Affiliations:** College of Life Sciences, Northeast Agricultural University, Harbin, 150030 China; State Key Laboratory of Reproductive Biology, Institute of Zoology, Chinese Academy of Sciences, Beijing, 100101 China

## Erratum to: Protein Cell 2015, 6(9):689–692 DOI 10.1007/s13238-015-0169-x

In the original publication of the article, the corresponding authors’ e-mail addresses were missed inadvertently. The corresponding authors’ details are provided in this erratum.

