# Supplementary material for: Generation of cell-type-specific gene mutations by expressing the sgRNA of the CRISPR system from the RNA polymerase II promoters
Source: Protein Cell. 2015 Jun 7;6(9):689–92. doi: 10.1007/s13238-015-0169-x (PMC4537475; doi:10.1007/s13238-015-0169-x)
Supplement: Supplementary file 1 — Supplementary material 1 (PDF 958 kb) [file 13238_2015_169_MOESM1_ESM.pdf]

Supplementary materials

This supplementary material file contains the following contents: Materials and methods, Supplementary Figures (S1~S2) and Supplementary Tables (S1~S2).

Materials and methods

Primers and vectors

Primers used in this paper is shown in Table S1. The commercial pEASY-T1-Simple cloning vector is bought from TransGen Company, the commercial pEGFP-C1, DsRed2-C1 and pAAV-Ef1a-DIO-mCherry-WPRE-pA vectors is offered by Embryo Biotechnology in College of life science in Northeast Agricultural University. The Cas9-2A-EGFP expression vector is offered by State Key Laboratory of Reproductive Biology in Institute of Zoology in Chinese Academy of Sciences.

Construction of vector

We amplified the SV40 polyA (pA) sequence (124 bp) with primers pA-F and pA-R from the commercial pEGFP-C1 plasmid, and synthesized a DNA sequence (125 bp, named BHBs, overlap 21 bp with pA sequence) containing multiple restriction endonuclease sites of *Bam*HI (1 site), *Kpn*I (1 site), *Hin*dIII (1 site), *Sac*I (1 site), and *Bsm*BI (2 site, with cohesive end of CTCG and GCCA respectively) with overlap primers BHBs-F and BHBs-R. Then the BHBs sequence was added to the 5′-end of pA sequence by overlapping PCR with primers BHBs-pA-F and pA-R (Fig. S1A). The resulted BHBs-pA sequence (226 bp) was cloned to the commercial pEASY-T1-Simple cloning vector.

We cloned the DsRed sequence (693 bp) with primers DsRed-F and DsRed-R from DsRed2-C1 vector with *Bsm*BI sites (Fig. S1B), chemical synthesized miR30-F-shRNA1, sgRNA targeting *p53* gene (sgp53) [14], and shRNA2-miR30-R sequences (Supplementary Material, and the shRNA targeted sequences,

ACAAGCTGGAGTACAACTACA and AAGATCCGCCACAACATCGAG are designed according to EGFP sequence) with *Bsm*BI sites by BGI Company, and performed Golden Gate cloning (1 μL BsmBI (NEB), 1 μL 10×NEBuffer 3.1 (NEB), 1 μL T7 ligase (3,000U/μL, NEB), 1 μL ATP (10mM, NEB), 1μL for each DNA fragment and 1 μL distilled water, total 10μL) in a thermocycler using the cycling conditions: 37 ºC for 5 minutes, 25 ºC for 5 minutes, for 30 cycles [17] (Fig. S1C). The resulted plasmid pEASYT1S-DsRed-miRsh-sgp53-pA was identified by *Dra*I restriction analysis (Fig. S1D).

The constitutive EF1a promoter (1178 bp) was amplified from pAAV-Ef1a-DIO-mCherry-WPRE-pA with primers EF1a-F and EF1a-R and the stem cell-specific mouse Oct4 gene promoter (mOct4P) (2177 bp) [18] was amplified from mouse genome DNA by nested-PCR (Fig. S1E). For the outer-PCR, 100 ng genome DNA was used in 50 μL PCR system containing 0.8 μM primers, 0.4 mM dNTP, 5 μL 10 × LA PCR Buffer II (Mg^{2+} plus), and 2.5 U LA Taq (TaKaRa); for the inner-PCR, the system was same to the outer-PCR except the template was 1 μL outer-PCR results. Melting temperature (Tm) for outer- and inner-PCR were both 65 ºC.

EF1a Promoter was sub-cloned to the final vector using *Bam*HI and *Hin*dIII restriction sites, and for mOct4P promoter the *Kpn*I and *Sac*I restriction sites were used. The final vectors pEASYT1S-EF1a-DsRed-miRsh-sgp53-pA and pEASYT1S-mOct4P-DsRed-miRsh-sgp53-pA were identified by *Pst*I restriction analysis (Fig. S1F) and Sanger sequencing.

Cell culture and transfection

mESCs cells were cultured as previously described [19] on mitomycin-C-treated MEFs cells with Dulbecco’s modified Eagle’s Medium (DMEM) (Gibco) plus 15% fetal bovine serum (FBS) (Gibco), 1,000 U/mL LIF (Chemicon), 2 mM glutamine (Sigma), 1 mM sodium pyruvate (Sigma), 0.1 mM β-mercaptoethanol (Sigma) and 0.1 mM non-essential amino acids at 37ºC with 5% CO_2 incubation. MEFs cells were isolated from E13.5 embryos and were cultivated with DMEM plus (Gibco) 10% FBS

(Gibco) at 37ºC with 5% CO_2 incubation.

MEFs cells were seeded into 6-well plates (Corning) one day prior to transfection at a density of 500,000 cells per well. Cells were transfected using Lipofectamine® LTX & PLUS™ Reagent (Life Technologies) according to the manufacturer's protocol. For each well of a 6-well plate a total of 2.5 μg plasmids (Cas9 : miRsh-sgp53 = 1:1) was used. mESCs cells were transfected by Neon™ Transfection System (Life Technologies) according to the manufacturer's protocol. One million cells and 20 μg plasmid (Cas9 : miRsh-sgp53 = 1:1) per each 100 μL Neon™ Tip were used.

T7EN1 Cleavage Assay and Sanger sequencing

GFP & DsRed double positive cells were sorted by fluorescence-activated cell sorting (FACS) at 48h after transfection and the genomic DNA was extracted by MicroElute Genome DNA kit (OMEGA). After amplified by LA Taq (TaKaRa), a total of 400ng of the purified PCR products were mixed with 2μl 10 × Taq polymerase PCR buffer (TaKaRa) and ultrapure water to a final volume of 20μl, and subjected to a re-annealing process to enable heteroduplex formation: 95ºC for 10 minutes, 95ºC to 85ºC ramping at -2ºC/s, 85ºC to 25ºC at -0.25ºC/s, and 25ºC hold for 1 minute [5]. Hybridized PCR products were digested with T7EN1 (NEB) for 30 min and separated by 2.5% agarose gel. For Sanger sequencing, PCR products were sub-cloned into pMD18-T vector (TaKaRa) and 20 bacterial plaques for each were sequenced by BGI Company.

Sequence of miR30-F-shRNA1:

CTCGACTAGGGATAACAGGGTAATTGTTTGAATGAGGCTTCAGTACTTTACA
GAATCGTTGCCTGCACATCTTGGAAACACTTGCTGGGATTACTTCTTCAGGT
TAACCCAACAGAAGGCTCGAAGAAGGTATATTGCTGTTGACAGTGAGCGCG
NNNNNNNNNNNNNNNNNNNNNNATAGTGAAGCCACAGATGTATNNNNNNN
NNNNNNNNNNNNNNNCATGCCTACTGCCTCGGACTTCAAGGG

Note: The miR30-F sequence is in bold italic font; the sense and antisense shRNA

targeting sequences are underlined.

Sequence of sgp53:

CTACGATCCTCGAGCTCCCTCTGAGCCGTTTAAGAGCTATGCTGGAAACAGCATAGCAAGTTTAAATAAGGCTAGTCCGTTATCAACTTGAAAAAGTGGCACCGAGTCGGTGCTTT

Note: The sgp53 targeting sequence are underlined.

Sequence of miR30-R:

GATCCAAGAAGGTATATTGCTGTTGACAGTGAGCGCGNNNNNNNNNNNNNNNNNNNNNNATAGTGAAGCCACAGATGTATNNNNNNNNNNNNNNNNNNNNNNCATGCCTACTGCCTCGGACTTCAAGGG***GCTACTTTAGGAGCAATTATCTTGTTTACTAAAACTGAATACCTTGCTATCTCTTTGATACATTTTTACAAAGCTGAATTAAAATGGTATAAATTAAATCACTTT***

Note: The miR30-R sequence is in bold italic font; the sense and antisense shRNA targeting sequences are underlined.

Supplementary Figures

Fig. S1

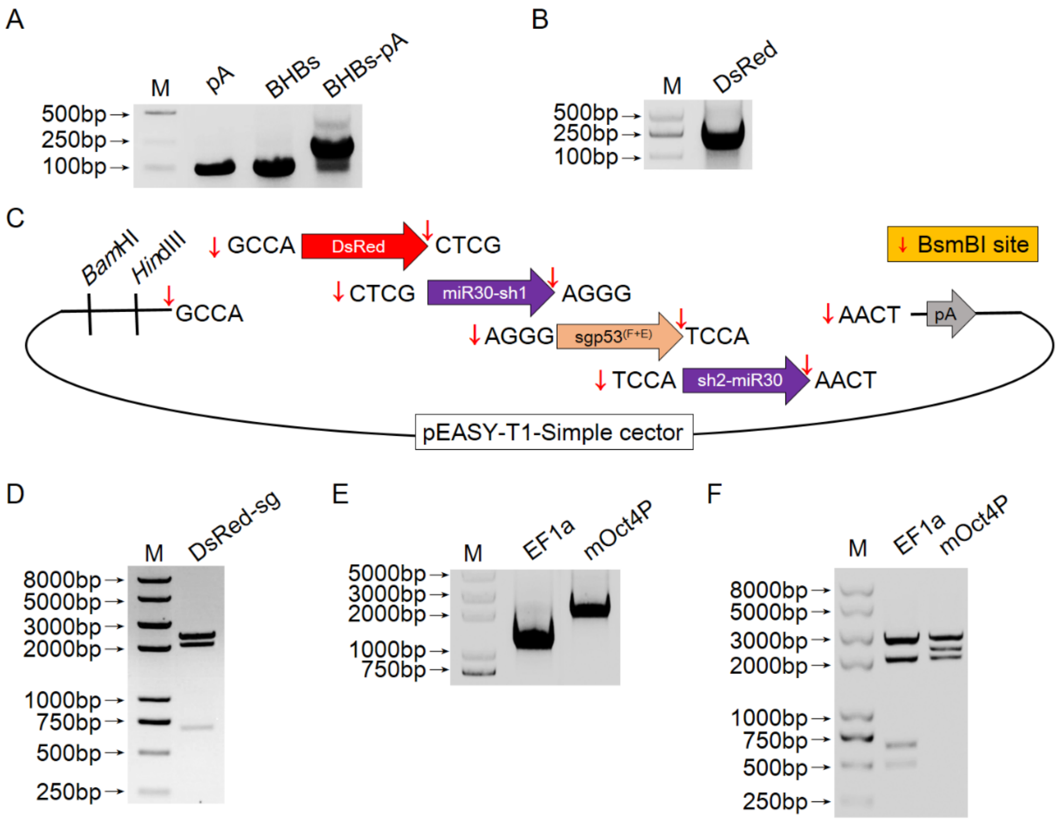

Figure S1. Construction of type II promoter-driving miRsh-sgp53 expression vector. (A) PCR and overlapping PCR amplification results of pA, BHBs, and BHBs-pA. The theoretical bands are 124 bp, 115 bp, and 218 bp. (B) PCR amplification result of DsRed. The theoretical band is 693 bp. (C) Golden Gate cloning technique for ligation of DsRed, miRsh-sgp53 cassette (the optimized sgRNA$^{(F+E)}$ backbone was used), and the SV40 polyA containing pEASY-T1-Simple vector. Restriction enzyme *Bsm*BI was used, and its cut sites are shown. *Bam*HI and *Hind*III sites are used for sub-cloning of type II promoters. (D) Restriction enzyme analysis of pEASYT1S-DsRed-miRsh-sgp53-pA by *Dra*I. Lane M stands for Trans2KplusII marker (TransGen); lane DsRed-sg stands for results of pEASYT1S-DsRed-miRsh-sgp53-pA with theoretical bands of 19 bp (invisible), 692 bp, 2183 bp, and 2407 bp. (E) PCR amplification results of EF1a promoter and mouse Oct4 promoter (mOct4P). The theoretical bands are 1178 bp (EF1a promoter), and

2177 bp (mOct4P). (F) Restriction enzyme analysis of final vectors pEASYT1S-EF1a-DsRed-miRsh-sgp53-pA and pEASYT1S-mOct4P-DsRed-miRsh-sgp53-pA by *Pst*I. Lane M stands for Trans2KplusII marker (TransGen); lane EF1a stands for results of pEASY-T1-S-EF1a-DsRed-miRsh-sgp53-pA with theoretical bands of 505 bp, 720 bp, 2221 bp, and 2991 bp; lane mOct4P stands for results of pEASY-T1-S-mOct4P-DsRed-miRsh-sgp53-pA with theoretical bands of 2221 bp, 2507 bp, and 2708 bp.

Fig. S2

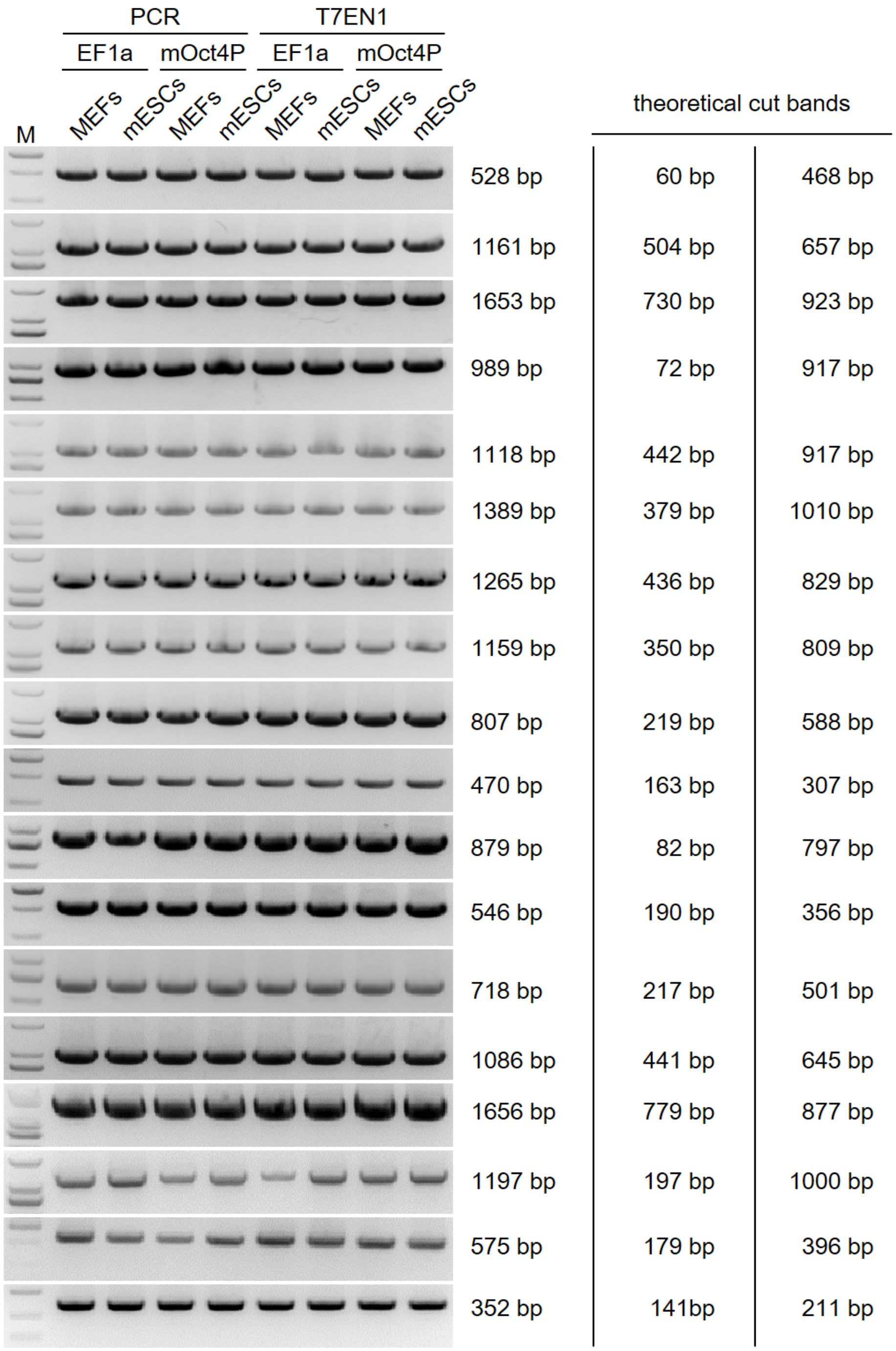

	PCR band	theoretical cut bands	
p53-off-4	528 bp	60 bp	468 bp
p53-off-7	1161 bp	504 bp	657 bp
p53-off-9	1653 bp	730 bp	923 bp
p53-off-12	989 bp	72 bp	917 bp
p53-off-13	1118 bp	442 bp	917 bp
p53-off-15	1389 bp	379 bp	1010 bp
p53-off-16	1265 bp	436 bp	829 bp
p53-off-18	1159 bp	350 bp	809 bp
p53-off-24	807 bp	219 bp	588 bp
p53-off-26	470 bp	163 bp	307 bp
p53-off-29	879 bp	82 bp	797 bp
p53-off-30	546 bp	190 bp	356 bp
p53-off-31	718 bp	217 bp	501 bp
p53-off-32	1086 bp	441 bp	645 bp
p53-off-33	1656 bp	779 bp	877 bp
p53-off-34	1197 bp	197 bp	1000 bp
p53-off-36	575 bp	179 bp	396 bp
p53-off-40	352 bp	141bp	211 bp

Figure S2. Off targeting analysis of partial potential site by T7EN1 assay. The PCR band and the theoretical cut bands are shown.

Supplementary Tables

Table S1. Primers used in this paper.

primer	Sequence (5′-3′)
pA-F	CAAACTTGTTTATTGCAGCTTATAATGGTTAC
pA-R	TAAGATACATTGATGAGTTTGGACAAACCAC
BHBs-F	GGATCCGGTACCGTGCCATTAGGGTGATGGTTCACGTAGTGGG CGTCAGTGTGAAAGCTTGAGCTCGCCATGAGA
BHBs-R	ATAAGCTGCAATAAACAAGTTTGAGACGATCAGTTATCTAGAT CCGGACGTCTCATGGCGAGCTCAAGCTTTCAC
BHBs-pA-F	GGATCCGGTACCGTGCCATTAGGGTGATGG
DsRed-F	CCGTCTCAGCCACCATGGCCTCCTCCGAGGACGTCATCAAG
DsRed-R	ACGTCTCACGAGGTCAGGAATTCCACAGGAACAGGTGGTGG
mOct4P-outer-F	ATGTCTCTTGTCCTGGCCAGTGAGTCACC
mOct4P-outer-R	AAGGCGAAGTCTGAAGCCAGGTGTC
mOct4P-inner-F	GGTACCGAATACAGACAGGACTGCTGGGCTGC
mOct4P-inner-R	GAGCTCCTGGAAAGACGGCTCACCTAGGGACG
EF1a-F	GGATCCGTGCCCGTCAGTGGGCAGAGCGCACATCG
EF1a-R	AAGCTTGGATACTCACGACACCTGAAATGGAAGAA
p53-test-F	CTCTTATTCTAACCTAAGTTCTGCCACG
p53-test-R	ACATAACAGACTTGGCTGTCCCAGA
p53-off-4-F	AGGTAGCACATCCCCACGTCAGTC
p53-off-4-R	AAGAGCGGCAGTCAAACATTAAACAGT
p53-off-7-F	GCCTTCAGTGGTTCATTCATTCATTC
p53-off-7-R	CACCTACCTATCCGTTCCTTCACCTC
p53-off-9-F	GAGACCTGAACTCGCAGCCCATT
p53-off-9-R	TTGCATCCAGCCCAACATTGTTATT
p53-off-12-F	TGTGTGTAGCAAGCACTTTCCCTCA
p53-off-12-R	TTCTCCTGCCTTCCTACGCACTCTA
p53-off-13-F	GAAGTGCCTGGGACTAAGTTTTCTGC
p53-off-13-R	AGCACGTGTTCTCTTGAGACGCATT
p53-off-15-F	GAACAATGGATATGGTTTTGAGCAGGT
p53-off-15-R	CGGCAATCGGTCCTCCAGACTAT
p53-off-16-F	GGGATGTGGTACAGATCATGCTAAGCT
p53-off-16-R	TGGTGTCTGGGTATGTTTGTTCAGACTAG
p53-off-18-F	CAATGGTAGAAGACTTGATTAGCATGCAC
p53-off-18-R	TTTAGTCCCAGAAGATTAAGACGGAAGTG
p53-off-24-F	TTCTCCCCAGAGTGATGTTGCTGC
p53-off-24-R	CCCGTGGTTTGACCTGAAATGTATG
p53-off-26-F	TTGCCTCACAGACCAAGACAAACATC
p53-off-26-R	ATTACAAGTCACGACCCCAAAGAACAG
p53-off-27-F	CCATATTGATCTGCATTGAGAACAAATG
p53-off-27-R	AAGCAGGACTCCACAAGCTACAGCT

p53-off-28-F	TTCAGGCATCTGAGACCTACGTAAGC
p53-off-28-R	TCACTCATTCCAGGTTACCATGGAGA
p53-off-29-F	ACTTCGTTCTGATGGTGGATGGGT
p53-off-29-R	CAGTCCTTGGGGATGACTGTTCG
p53-off-30-F	ACTCACAGCTCTGAAGCAAACTGTCC
p53-off-30-R	GTGCTTGATATGGCCCATAGGTCA
p53-off-31-F	CGTCTCAAGTAGTTGAACCACAGCG
p53-off-31-R	TGGCCTGTGTAACCTGGGTAGTACC
p53-off-32-F	CATGGGGCGTTGCTTCACTCA
p53-off-32-R	CAATCTCTGTGTCCATTGAGGTGGTC
p53-off-33-F	TGAGAAAAGGGTGTTGGGACTGG
p53-off-33-R	GGAACCTGATGGTGATGGGGAG
p53-off-34-F	CACCACTAGGAGCACGAGTGTGACA
p53-off-34-R	CGTTTGGAGGAAGAAGTTGAAGTGAGT
p53-off-36-F	TTCGCACTTAGTCAAGCTGGAAACAC
p53-off-36-R	GCTCACCATGTCCTCGTTTCATTGT
p53-off-40-F	GCTGAGACTCTGCTGGACTGGAAAG
p53-off-40-R	ATTCACCTGCCCAGAGTCAGGATC

Table S2. Potential off targets for sgp53.

Name	chr	strand	start	end	SEEDPAM
p53-off-1	chr1	+	135100382	135100396	gggccaatTCCCTCTGAGCCAGG
p53-off-2	chr2	-	57777462	57777476	gtctcccaTCCCTCTGAGCCTGG
p53-off-3	chr2	-	117502971	117502985	aactttgtTCCCTCTGAGCCAGG
p53-off-4	chr2	+	119242394	119242408	cactcatCTCCCTCTGAGCCAGG
p53-off-5	chr2	+	162580126	162580140	accctcgaTCCCTCTGAGCCTGG
p53-off-6	chr2	+	178128354	178128368	cagacactTCCCTCTGAGCCAGG
p53-off-7	chr3	-	51396547	51396561	ctggctcCTCCCTCTGAGCCTGG
p53-off-8	chr3	-	117845564	117845578	gcaatcagTCCCTCTGAGCCAGG
p53-off-9	chr4	-	135468138	135468152	ctcccttCTCCCTCTGAGCCCGG
p53-off-10	chr4	+	136712541	136712555	cttggggtTCCCTCTGAGCCAGG
p53-off-11	chr4	+	139157277	139157291	ctgagacaTCCCTCTGAGCCAGG
p53-off-12	chr5	+	32347379	32347393	acccagtCTCCCTCTGAGCCTGG
p53-off-13	chr5	-	64226752	64226766	caaaggcCTCCCTCTGAGCCAGG
p53-off-14	chr5	+	67323688	67323702	ttccttctTCCCTCTGAGCCTGG
p53-off-15	chr5	-	101930696	101930710	aggagatCTCCCTCTGAGCCAGG
p53-off-16	chr5	-	108554174	108554188	ctaggttCTCCCTCTGAGCCTGG
p53-off-17	chr5	+	119592980	119592994	ccactgtgTCCCTCTGAGCCAGG
p53-off-18	chr5	-	136687931	136687945	aagaattCTCCCTCTGAGCCAGG
p53-off-19	chr5	-	140008543	140008557	aagtcccaTCCCTCTGAGCCGGG
p53-off-20	chr7	-	48083496	48083510	taccacatTCCCTCTGAGCCGGG
p53-off-21	chr8	-	24677195	24677209	ctgcttctTCCCTCTGAGCCAGG
p53-off-22	chr8	+	70794745	70794759	aggtacatTCCCTCTGAGCCTGG
p53-off-23	chr8	+	121143176	121143190	ccttgcatTCCCTCTGAGCCTGG
p53-off-24	chr9	+	14789773	14789787	ggaacacCTCCCTCTGAGCCTGG
p53-off-25	chr9	-	50509204	50509218	atactgctTCCCTCTGAGCCTGG
p53-off-26	chr10	-	115472725	115472739	acaggctCTCCCTCTGAGCCAGG
p53-off-27	chr11	+	85638263	85640276	atgcctttTCCCTCTGAGCCTGG
p53-off-28	chr11	-	114927930	114927944	tagaaatCTCCCTCTGAGCCAGG
p53-off-29	chr12	-	53922240	53922254	ggcttcGCTCCCTCTGAGCCCGG
p53-off-30	chr13	-	20554333	20554347	acacttcCTCCCTCTGAGCCAGG
p53-off-31	chr13	-	43182889	43182903	tgaaagGCTCCCTCTGAGCCTGG
p53-off-32	chr13	+	59874447	59874461	tctgagagTCCCTCTGAGCCAGG
p53-off-33	chr14	-	24179178	24179192	ttccgcctTCCCTCTGAGCCTGG
p53-off-34	chr14	+	57053101	57053115	tcagggtCTCCCTCTGAGCCTGG
p53-off-35	chr14	-	104819248	104819262	gagaccctTCCCTCTGAGCCTGG
p53-off-36	chr15	+	8883331	8883345	cattcattTCCCTCTGAGCCAGG
p53-off-37	chr15	+	34684633	34684647	aagctacaTCCCTCTGAGCCTGG
p53-off-38	chr15	+	80408089	80408103	gtcccataTCCCTCTGAGCCTGG
p53-off-39	chr16	+	32681861	32681875	aagaaagtTCCCTCTGAGCCAGG
p53-off-40	chr18	+	75458672	75458686	tggggtaCTCCCTCTGAGCCTGG
p53-off-41	chrX	+	112905517	112905531	gcagcaaaTCCCTCTGAGCCTGG

Note: SEEDPAM means the "seed sequence" at the 3′ end of the sgRNA and the PAM sequence NGG.
